# A risk signature based on necroptotic-process-related genes predicts prognosis and immune therapy response in kidney cell carcinoma

**DOI:** 10.3389/fimmu.2022.922929

**Published:** 2022-09-16

**Authors:** Jingxian Li, Xun Liu, Yuanjiong Qi, Yang Liu, E. Du, Zhihong Zhang

**Affiliations:** Tianjin Institute of Urology, The Second Hospital of Tianjin Medical University, Tianjin, China

**Keywords:** RCC, necroptosis, tumor immune microenvironment, prognostic signatures, immune therapy

## Abstract

Necroptosis is a regulated form of cell necroptotic process, playing a pivotal role in tumors. In renal cell cancer (RCC), inhibiting necroptosis could promote the proliferation of tumor cells. However, the molecular mechanisms and prognosis prediction of necroptotic-process-related genes in RCC are still unclear. In this study, we first identified the necroptotic process prognosis-related genes (NPRGss) by analyzing the kidney renal clear cell carcinoma (KIRC) data in The Cancer Genome Atlas (TCGA, n=607). We systematically analyzed the expression alteration, clinical relevance, and molecular mechanisms of NPRGss in renal clear cell carcinoma. We constructed an NPRGs risk signature utilizing the least absolute shrinkage and selection operator (LASSO) Cox regression analysis on the basis of the expression of seven NPRGss. We discovered that the overall survival (OS) of KIRC patients differed significantly in high- or low-NPRGs-risk groups. The univariate/multivariate Cox regression revealed that the NPRGs risk signature was an independent prognosis factor in RCC. The gene set enrichment analysis (GSEA) and gene set variation analysis (GSVA) were used to explore the molecular mechanisms of NPRGss. Immune-/metabolism-related pathways showed differential enrichment in high-/low-NPRGs-risk groups. The E-MTAB-1980, TCGA-KIRP, GSE78220, the cohort of Alexandra et al., and IMvigor210 cohort datasets were respectively used as independent validation cohorts of NPRGs risk signature. The patients in high- or low-NPRGs-risk groups showed different drug sensitivity, immune checkpoint expression, and immune therapy response. Finally, we established a nomogram based on the NPRGs risk signature, stage, grade, and age for eventual clinical translation; the nomogram possesses an accurate and stable prediction effect. The signature could predict patients’ prognosis and therapy response, which provides the foundation for further clinical therapeutic strategies for RCC patients.

## Introduction

As the third most commonly diagnosed urological cancer, renal cell carcinoma (RCC) causes over 179,000 deaths every year worldwide (1). Approximately 70% of patients are diagnosed with kidney renal clear cell carcinoma (KIRC). KIRC is a highly immuno-invasive tumor (2). Several studies have reported that renal cancer progression is correlated with the disturbance of the tumor’s metabolism and immune microenvironment (3). Although a range of approaches such as surgery, chemotherapy, targeted therapy, radiotherapy, and recently immunotherapy has been used in the clinical treatment of KIRC, the efficacy of these agents is still limited (4, 5). Therefore, KIRC is still one of the most challenging clinical problems. It is necessary to find new therapeutic targets for KIRC.

Necroptosis is a tightly regulated cell death (RCD) mimicking the morphological features of necrosis (6), which is primarily mediated by receptor-interacting protein kinase 1 (RIPK1), receptor interacting serine/threonine kinase 3 (RIPK3), and mixed lineage kinase domain-like pseudokinase (MLKL) and characterized to be inhibited by the necrostatin-1 (Nec-1). Necroptosis plays an important role in regulating cancer biologies, such as oncogenesis, cancer metastasis, cancer subtypes, and cancer immunity (3). The necroptosis-related pathway and core regulators are correlated with metabolic signaling and cancer immune surveillance. Targeting necroptosis *via* a series of compounds, drugs, and agents inducing or manipulating the necroptosis-related pathway has also emerged as a novel approach for bypassing apoptosis resistance and supporting antitumor immunity in cancer therapy. Necroptosis is considered to play an important role in renal carcinoma cells. Necroptosis resistance has been reported to promote the proliferation of renal carcinoma cells (7). High expression of RIPK1/3 in KIRC tumor cells increases the sensitivity of tumor necrosis factor alpha 1 (TNF-1) receptor-induced necroptosis (8). Exploring the potential function, molecular mechanism, and clinical relevance of necroptosis in KIRC could provide a theoretical foundation for subsequent target therapy.

This study identified necroptotic process prognosis-related genes (NPRGss) and explored the molecular mechanism and clinical relevance of these genes in KIRC. Although previous research has constructed the necroptosis-related genes risk signature in KIRC, these models still have limitations in predicting prognosis and guiding clinical treatment (9). Herein, we constructed a novel seven genes risk signature called NPRGs risk signature based on the expression of NPRGss in The Cancer Genome Atlas Kidney Renal Clear Cell Carcinoma (TCGA-KIRC). We found that the prognosis of the patients, immune checkpoint expression, gene ontology, and pathways enrichment were significantly different in high- or low-NPRGs-risk groups. The univariate and multivariate Cox regression analyses reveal that the NPRGs risk signature was an independent prognostic factor for KIRC. Furthermore, we found that the IC50 of drugs targeting KIRC showed a significant difference in the high- or low-NPRGs-risk groups, revealing that the patients in different NPRGs-risk groups have various sensitivities to drugs. The NPRGs risk signature was validated in E-MTAB-1980 and TCGA-KIRP cohorts, suggesting that this signature is practical in predicting the prognosis for RCC. In KIRC and KIRP cohorts, we found the that NPRGs risk in patients with progressive disease (PD) is higher. In three independent immune therapy cohorts, the patients of the high-NPRGs-risk group are more accessible to SD/PD. Finally, we constructed a nomogram model based on the NPRGs risk signature and other clinical variables. The nomogram showed an accurate and stable forecasting ability for patients with 3-/5-/7-year overall survival. These results revealed that NPRGs risk signature could predict prognosis and immune therapy response for tumor patients. In conclusion, our study revealed the role of NPRGss in KIRC and constructed a feasible risk signature to predict patients’ prognosis and therapy response.

## Materials and methods

### Identification of necroptotic-process-related genes and acquisition of public data

To identify necroptotic-process-related genes, we referred to high-quality articles (10) and a public database (https://www.ebi.ac.uk/QuickGO/) (11). Thirty-six genes were determined. The information on genes is shown in **Supplementary Table S1**. The gene RNA-seq expression profiles of KIRC and adjacent normal tissues were downloaded from TCGA (https://xena.ucsc.edu/) and Array Express database (https://www.ebi.ac.uk/arrayexpress). The TCGA-KIRC includes 535 tumors and 72 normal samples. E-MTAB-1980 dataset includes 101 RCC samples with exactly clinical survival information. The TCGA-KIRP includes 285 tumor samples. The TCGA-KIRC and TCGA-KIRP mRNA expression value was normalized and transformed to a CPM unit utilizing the edgeR packages. Furthermore, patients’ clinical information from the TCGA-KIRC and E-MTAB-1980 were respectively collected for subsequent analysis.

Then, we downloaded three external cohorts with immune therapy, including GSE78220 (12), IMvigor210 (13), and the UC cohort of Alexandra et al. (14), for subsequent immune therapy response validation. The edge packages normalized the IMvigor210 cohorts. The read counts data of GSE78220 and cohorts of Alexandra et al. were transformed to log2(TPM+1) unit. The GSE78220 dataset includes 28 anti-PD-1 therapy melanoma patients, the IMvigor210 includes 348 anti-PD-1 therapy patients with different types of cancer, and the cohort of Alexandra et al. includes 25 anti-PD-1 therapy urothelial cancer patients.

The lncRNA annotation data were obtained from the Gencode database (https://www.gencodegenes.org/human/). The highly conserved microRNA family data were downloaded from the miRcode database to conduct microRNA and lncRNA target prediction (http://www.mircode.org/).

### Differential expression analysis and identification of NPRGss

The differential expression genes (DEGs) between two groups were screened using the *limma* R package downloaded from the Bioconductor (https://www.bioconductor.org/). We have adjusted the p-value by the Benjamini–Hochberg method to control the false discovery rate (FDR). The gene with an adjusted p-value (FDR)<0.05 and a |log2(fold change)| > 0.5 was regarded as DEG. To determine the NPRGss, we matched the TCGA-KIRC clinical information and mRNA expression data of genes. Then, we conducted the univariate Cox progression analysis by utilizing the *coin* R-package. The prediction performance was evaluated using the hazard regression model. We further used the *VennDiagram* R-package to depict a Venn diagram to represent the overlapping genes between the DEGs and prognosis-related genes. These overlapping genes were defined as NPRGss. A p-value<0.05 was considered statistically significant difference. To validate the expression alteration of NPRGs, we downloaded the immunohistochemistry data from the Human Protein Atlas (HPA) database (https://www.proteinatlas.org/) for further validation. This database currently contains 44 human tissue protein data, and the protein data cover 15,323 genes for which there are available antibodies. In the selection of protein immunohistochemistry, we selected data from the same antibody and tried to select the same patient on this basis.

### Survival prediction verification

The Kaplan–Meier survival curves were portrayed using *survminer* and *survival* R-package. The ideal cutoff point of NPRGs expression was determined by X-tile software (15). The relationship between different objects and patient survival was estimated by applying the log-rank test.

### The cross-talk of NPRGss

The Pearson correlation coefficient (PCC) among genes was calculated by applying the *Hmisc* R-package to explore the cross-talk between the NPRGss. Then, we used the *corrplot* R-package to depict the correlation coefficient diagram. To understand the potential target, we respectively presented NPRGss to the STRING database (https://string-db.org/cgi/input.pl), and then, the protein–protein interaction (PPI) network was visualized by the Cytoscape software.

### Analysis of gene set enrichment and gene set variation analysis

The dataset of the cancer-related hallmark pathways, cell components, biological process, molecular functions, and Kyoto Encyclopedia of Genes and Genomes (KEGG) pathways were downloaded from the GSEA website (https://www.gsea-msigdb.org/gsea/index.jsp). The TCGA-KIRC expression dataset was subjected to the gene set variation analysis (GSVA) to calculate the activity score of different terms. The relationship between the NPRGss expression and activity scores was estimated by calculating the Pearson correlation coefficient (PCC). The |PCC| > 0.3 and p-value< 0.05 were considered moderately correlated. The |PCC| > 0.6 and p-value< 0.05 were considered as highly correlated.

### Construction and validation of a NPRGss risk signature

The patients were randomly divided into the training and testing cohorts with a ratio of 1:1 using the “*createDataPartition*” R-function. Then, the LASSO-penalized Cox (LASSO-Cox) regression analysis was carried out to rule out genes with an overfitting tendency and construct a prognosis-related signature with the *glmnet* R-package. In the TCGA training cohort, the characteristic gene signatures were established by the LASSO-Cox regression analysis. The optimal penalty parameter l that correlated with the minimum 10-fold cross-validation was selected to screen the signatures. The lambda value with the minimum mean square error was used to reduce the prediction error of the model. Then, we used the coefficients obtained from the LASSO-Cox regression algorithm and gene expression level to yield a risk score. The risk-score equation was shown as follows:


$${\text{Riskscore= sum (Exp}}_{\text{gene}}*\text{coef)}$$

Meanwhile, we also use the TCGA-KIRC testing cohort, E-MTAB-1980 cohort, and TCGA-KIRP cohort to verify the risk model. Receiver operating characteristic (ROC) curve analyses were performed to estimate the accuracy of models by employing the survivalROC R-package. Kaplan–Meier survival curve demonstrated the prognosis difference between high- and low-risk cohorts. The univariate and multivariate Cox regression analyses were carried out for risk scores and clinical variables. The variables with p<0.05 in both univariate and multivariate Cox regressions were considered independent risk factors.

### Molecular mechanism in high- or low-NPRGs-risk groups

The TCGA-KIRC cohort was divided into high- or low-NPRGs-risk groups based on the median NPRGs risk score. We first calculated the DEGs between the high- and the low-risk group. The gene with |logFC|>0.5 and adjusted p-value was regarded as expressed differently. Then, all DEGs were ranked by logFC and subjected to *clusterProfiler* R-package to conduct a pre-ranked gene set enrichment analysis (GSEA). The top 10 significant enrichment terms of hallmark pathways, KEGG pathways, and Gene Ontology (GO) (including cell component, biological progression, and molecular functions) were demonstrated.

### Analysis of immune infiltration and metabolic reprogramming

The immune score, tumor purity, and stromal score of each patient were estimated by *estimate* R-package. The gene sets representing 24 immune cell types across tumors were obtained from the published research (16). Then, the single-sample gene set enrichment (ssGSEA) method in *GSVA* R-package was carried out to quantitate the infiltration levels of these gene sets. The ssGSEA score of individual immune cell types was standardized by the equation of our previous study (17). The proportion of 22 immune cell types for an individual sample was computed by Cell Type Identification by Estimating Relative Subsets of RNA Transcripts (CIBERSORT). We further obtained the metabolic signature gene sets from the previous study (18), which include carbohydrate metabolism (286 genes), amino acid metabolism (348 genes), nucleotide metabolism (90 genes), tricarboxylic acid cycle (TCA cycle, 148 genes), integrated energy metabolism (110 genes), lipid metabolism (766 genes), and vitamin cofactor metabolism (168 genes). Then, we evaluated the activity score of the metabolic signatures for each sample using the ssGSEA method. The correlation between NPRGss and metabolic signature was calculated by the Pearson method.

### Drug sensitivity analysis

The DEGs between the high- and the low-NPRGs-risk group were subjected to public drug prediction (https://design-v2.cancerresearch.my/query) (19). A ranked-based list of inhibitors is generated, and every drug has a connectivity score. A connectivity score closer to 1 indicates that the drug has the most excellent efficacy, and closer to −1 indicates that the drug has minimal efficacy. To analyze the target drug sensitivity of the patients, we identified the targeting drugs treated for KIRC patients from published literature (20). Then, we obtained the large-scale gene expression (GDSC1_Expr) and drug screening data (GDSC1_Res) as a training cohort to build ridge regression models. The models were applied to TCGA-KIRC datasets (testing cohort) to yield drug sensitivity predictions for each patient using the oncoPredict R Packages (21). Through this analysis, we determined the IC50 value that indicated each patient’s drug sensitivity and compared it in high- or low-NPRGs-risk groups.

### Construction of nomogram

The variables (including age, gender, grade, stage, and risk score) that correlated with the prognosis of patients estimated by univariate Cox regression analysis in TCGA-KIRC, E-MTAB-1980, and TCGA-KIRP cohorts were subjected to multivariate Cox regression analysis. Due to the lack of grade data in TCGA-KIRP, the variables (including age, gender, stage, and risk score) were used to construct the analysis. The stepwise regression method based on the minimum of Akaike information criterion was utilized to screen variables. Then, we utilized the rms R-package to construct a nomogram to evaluate the probability of 3-/5-/7-year overall survival (OS). The area under the time-dependent receiver operating characteristic curve (time-dependent AUC), concordance index (C-index), and calibration plots were utilized to estimate the discriminative accuracy. The AUC values and C-index >0.7 were regarded as reasonable estimations. Then, we depicted the decision curve analysis (DCA) to estimate the clinical benefits of the nomogram.

### Statistical analysis

The unpaired t-test analysis was performed to compare the differences between the two groups. The log-rank test was utilized to evaluate prognosis differences. The chi-square test was used to test the constituent ratio differences between different groups. The univariate/multivariate Cox regression analysis was employed to estimate the relationship between variables and patients’ prognoses. The Pearson method was calculated to evaluate the correlation between the two groups.

## Results

### Identification of NPRGss

**Figure 1:**
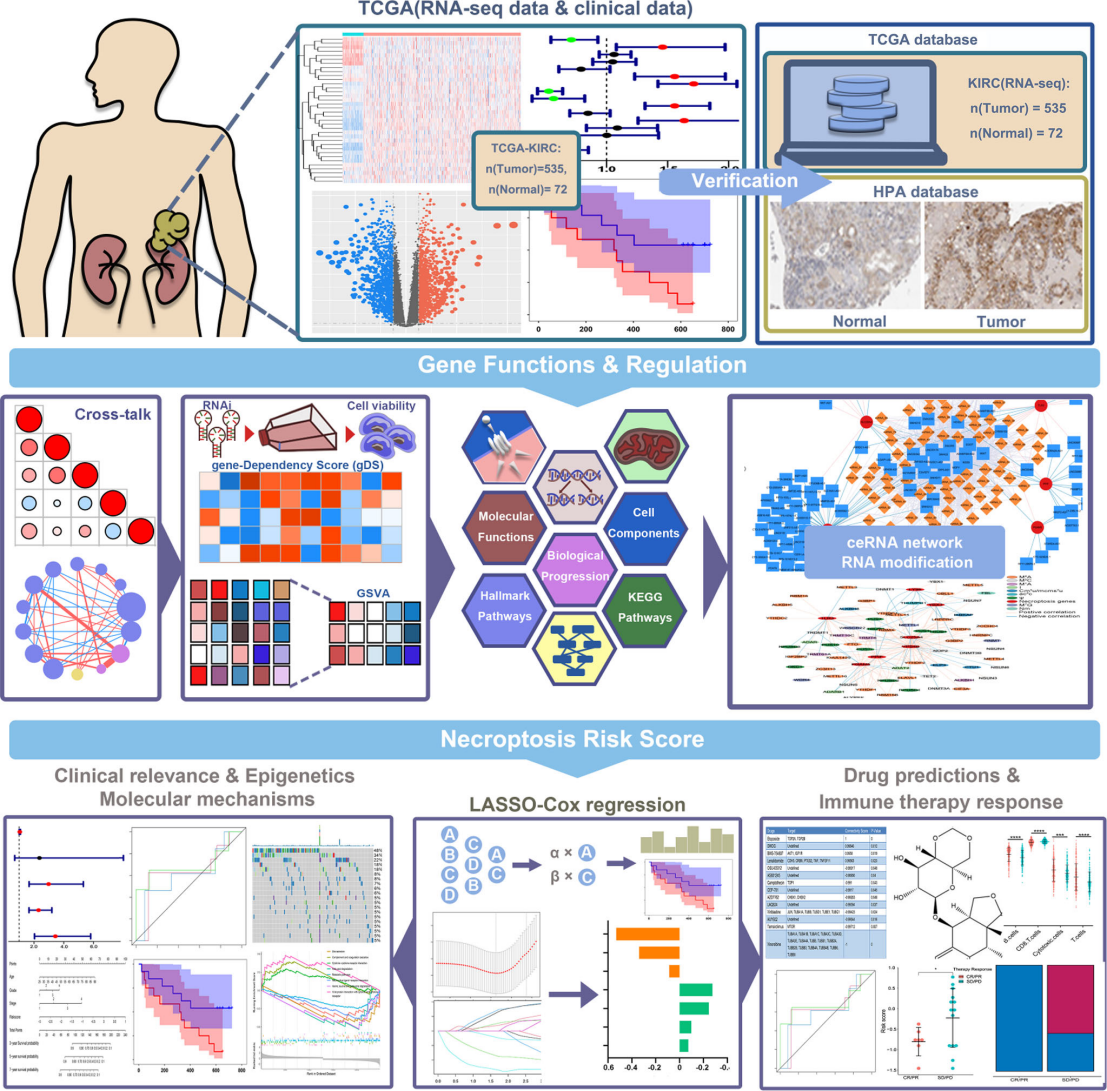
Flow chart of the research.

**Figure 2:**
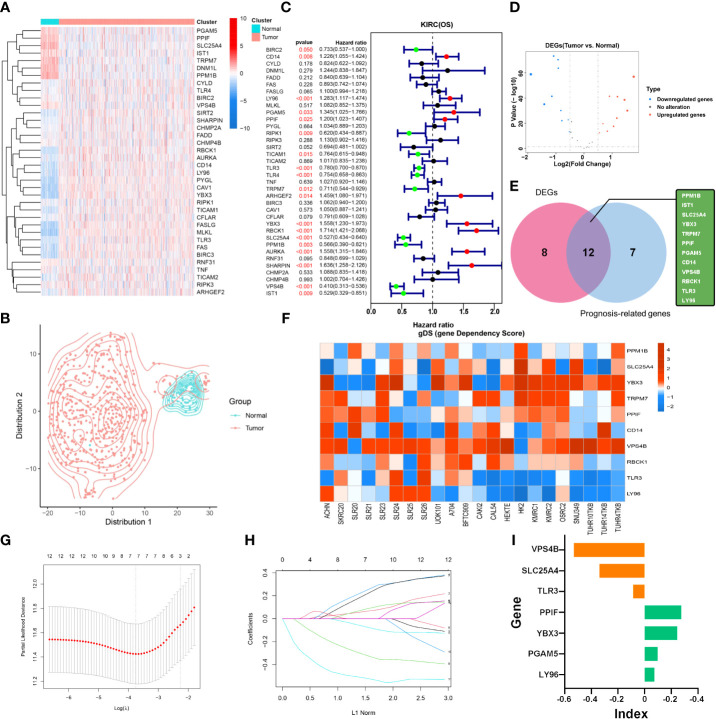
Identification of NPRGss and NPRGs risk signature. **(A)** Heatmap showing the expression of necroptotic-process-related genes. **(B)** The t-SNE analysis in tumor and normal groups. **(C)** The forest plots were depicted to show the prognosis-related genes determined by the univariate Cox regression analysis. **(D)** The volcano figure shows the necroptotic-process-related DEGs. **(E)** Venn diagram showing the overlapping NPRGss. **(F)** Heatmap showing the gDS (gene dependency score) of NPRGss in a renal cancer cell. **(G, H)** LASSO regression analyses of NPRGss using the OS model. **(I)** The bar chart displays LASSO regression coefficients of the NPRGss.

The specific workflow of this study is shown in **Figure 1**. First, we determined 36 necroptotic process-related genes from the previous paper and database (**Supplementary Table S1**). The expression of these genes could significantly distinguish the tumor and normal tissues (**Figures 2A, B**). Nineteen (51.35%) genes were related to the OS of KIRC patients (**Figure 2C**), and 20 (55.56%) genes showed differential expression between KIRC and normal tissues (**Figure 2D**). We ultimately determined 12 overlapping genes through intersection analysis and named them necroptotic process prognosis-related genes (NPRGss) (**Figure 2E**). Among these, CD14, LY96, RBCK1, YBX3, and TLR3 showed upregulated tumors compared to adjacent normal tissues (**Supplementary Figure S1A**). IST1, PGAM5, PPM1B, SLC25A4, PPIF, VPS4B, and TRPM7 showed downregulated tumors compared to adjacent normal tissues. The alteration of these gene-encoding protein levels was verified on the HPA database (**Supplementary Figure S1B**). The results showed that the expression alteration of CD14, TLR3, IST1, SLC25A4, PGAM5, and YBX3 at the protein level was consistent with its expression at RNA-seq. However, the PPM1B and VPS4B showed no difference, and other gene protein expression data cannot be found in the HPA database. We determined the ideal cutoff of 12 NPRGss expressions by X-tile software (15) and ulteriorly depicted the Kaplan–Meier survival curve (**Supplementary Figure S2**). The result displayed that the high expression of YBX3, PPIF, PGAM5, CD14, RBCK1, and LY96 were related to poor outcomes in KIRC patients. On the contrary, the patients with low expression of PPMIB, IST1, SLC25A4, TRPM7, VPS4B, and TLR3 showed a worse prognosis. Meanwhile, we used gene dependency score (gDS) to estimate the “essentiality” of NPRGss (22). gDS< −1 of genes indicated that the deletion severely affects cell viability. Genes with gDS > 0 indicated that the deletion has no effect on cell viability. Genes with gDS between −1 and 0 indicated that the deletion influences cell viability to some extent but is not fatal. Our results showed that the deletion of risk genes, such as TLR3 and LY96, were lethal for kidney cancer cells (**Figure 2F**). On the contrary, some protective genes, such as TRPM7 and VPS4B, were not affected in most cells.

To further detect the contribution of NPRGss for KIRC patients, we established an NPRGs signature using the LASSO Cox regression analysis based on OS. The TCGA-KIRC cohort was divided into the TCGA training and testing cohorts at 1:1. Then, a prognostic model was established applying LASSO Cox regression analysis based on the expression values of 12 NPRGss in the TCGA training cohort (**Figures 2G, H**). Ultimately, seven NPRGss, namely, LY96, PGAM5, PPIF, TLR3, YBX3, SLC25A4, and VPS4B, were identified. The coefficients obtained by the LASSO algorithm were used to determine the NPRGs signatures of the KIRC training and testing cohorts. The risk score was calculated as follows (**Figure 2I**):


$$\begin{array}{l} NPRGss riskscore = LY96 * 0.0726 +PGAM5 * 0.0987+ PPIF * 0.2752 -TLR3 * 0.0865 + YBX3 * 0.2452 -SLC25A4 * 0.3386 -VPS4B * 0.5309 \end{array}$$

### The cross-talk and mechanisms of NPRGss

To explore the cross-talk of seven NPRGss in KIRC, the Pearson correlation coefficient (PCC) among the mRNA expression of these genes was calculated (**Supplementary Figure S3A**). We observed that different genes correlated negatively or positively with other genes. PPIF showed a highly positive correlation with PGAM5 and SLC25A4. SLC25A4 showed a highly positive correlation with PPIF, PGAM5, and VPS4B. TLR3 showed a highly negative correlation with PGAM5 and PPIF. LY96 showed a highly negative correlation with SLC25A4. We further explored the potential target proteins directly interacting with these genes based on the STRING interaction database (**Supplementary Figure S3B**, details in **Supplementary Table S2**). We found that SLC25A4 interacted with PPIF, TLR3 interacted with LY96 and PGAM5, and they have the same target proteins. However, VPS4B and YBX3 have no interaction with other NPRGss. Then, the underlying mechanisms of NPRGss in KIRC were explored by calculating the activity score of different cancer-related hallmark pathways, GO terms (including cell components, biological progressions, and molecular functions), and KEGG pathways. The PCCs between seven NPRGss expressions and these activity scores were further computed, and the correlation with |PCC|≥0.6 and adjusted p-value< 0.5 were depicted by the network diagram (**Supplementary Figure S3C**, details in **Supplementary Table S3**). The results showed that LY96, VPS4B, SLC25A4, and PPIF were highly correlated with multiple GO terms and KEGG pathways. Interestingly, we discovered that more GO terms and KEGG pathways were correlated with the expression of LY96, revealing that LY96 may play an essential role in regulating KIRC development. Meanwhile, we conducted the same analysis between seven NPRGss and hallmark-related cancer pathways. The results showed that different genes might be involved in various pathways (**Supplementary Figure S3D**, details in **Supplementary Table S3**). For example, the expression of LY96 showed a positive correlation with the activity of apoptosis, complement, and inflammatory response-related pathways. The expression of SLC25A4 showed a negative correlation with most hallmark pathways. In summary, different NPRGss possess different functions and involve different mechanisms, which provided a foundation for further experiments about NPRGss in regulating KIRC. We analyzed the expression of NPRGss and lncRNA in KIRC to explore NPRGs-related regulatory factors. The interactions with |PCC|>0.5 are shown in **Supplementary Figure S4A**. Meanwhile, we downloaded the highly conserved microRNA families from the mircode to predict the potential miRNA that interacted with lncRNA. A ceRNA network between lncRNA–miRNA–NPRGss was established (**Supplementary Figure S4A**, details in **Supplementary Table S4**). The result showed that VPS4B and TLR3 have an abundant regulatory network. However, YBX3 and LY96 have only a small amount of regulation. Furthermore, RNA modification is also associated with KIRC in the published literature (23, 24). Hence, we collected genes associated with RNA methylation and calculated the correlation between RNA regulators and NPRGss expression (**Supplementary Table S5**). As shown in **Supplementary Figure S4B**, NPRGss is highly associated with multiple RNA regulator, revealing that these regulators may regulate NPRGss in RNA expression.

### The prognosis prediction of NPRGs risk signature

**Figure 3:**
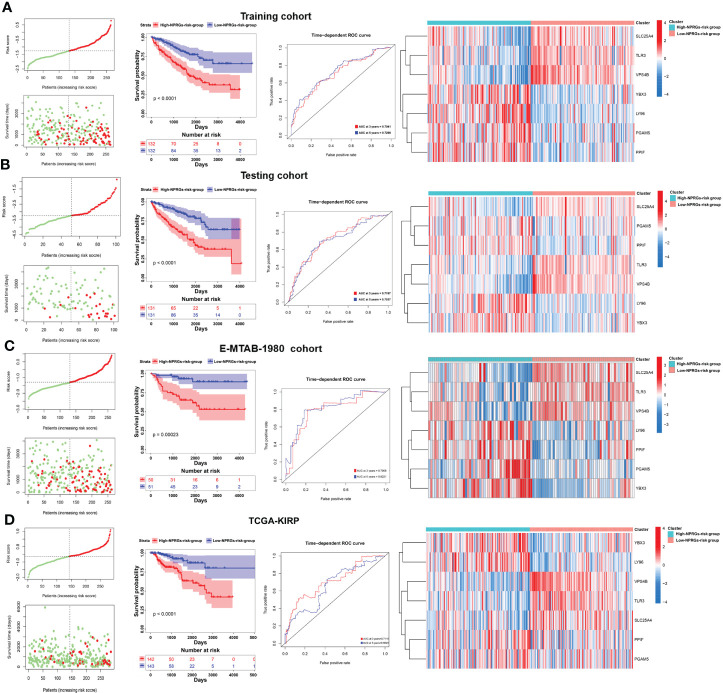
Prognostic analyses of NPRGs risk signature in TCGA-KIRC training cohort, TCGA-KIRC testing cohort, E-MTAB-1980 cohort, and TCGA-KIRP cohort. **(A)** Distribution of OS, OS status, and risk score in the TCGA-KIRC training cohort; the Kaplan–Meier curves of the high- or low-NPRGs-risk cohort in TCGA-KIRC training cohort; AUC of time-dependent ROC curves was used to evaluate the prognostic performance of the NPRGs risk score; the heatmap of the expression of NPRGss in high- or low-NPRGs-risk groups. **(B–D)** The same analyses in the TCGA-KIRC testing cohort, E-MTAB-1980 cohort, and TCGA-KIRP cohort.

To further explore the predictive ability of NPRGs risk signature in patients’ prognosis, we stratified the KIRC training cohort into a high- or low-NPRGs-risk groups according to the median risk score. In the training cohort, the number of death increased, accompanied by the increasing risk score (**Figure 3A**). The patients of the high-NPRGs-risk group showed a worse prognosis than those of the low-NPRGs-risk group. The time-dependent AUC showed a discriminative accuracy in 3- and 5-year survival (3-year AUC = 0.7041, 5-year AUC = 0.7290). We further divided the subgroups according to clinical features of TCGA-KIRC. Patient outcomes with different risk scores were assessed in different subgroups, and the results showed that in most subgroups of clinical features, patients with high NPRGs risk showed worse prognosis than those with low NPRGs risk, suggesting that the NPRGs risk signature has strong robustness (**Supplementary Figure S5A**). The same study was performed in the TCGA-KIRC validation cohort and an external validation cohort (E-MTAB-1980) (**Figures 3B, C**). The results indicated that the NPRGs risk signatures showed discrimination performance in 3- and 5-year survival. Intriguingly, the expression of YBX3, LY96, PGAM5, and PPIF were higher in the high-NPRGs-risk group than in the low-NPRGs-risk group, which is consistent in TCGA training, TCGA validation, and E-MTAB-1980 cohort, suggesting that high co-expression of these genes promotes the poor outcome of KIRC patients. We apply the NPRGs risk signature to TCGA-KIRP cohort. Excitingly, the NPRGs risk signature also showed a feasible prediction performance in the KIRP cohort (**Figure 3D**), indicating that the NPRGs risk signature can be applied to the prognostic prediction of most RCC patients not just KIRC.

### Clinical relevance and mechanisms based on NPRGs signature

**Figure 4:**
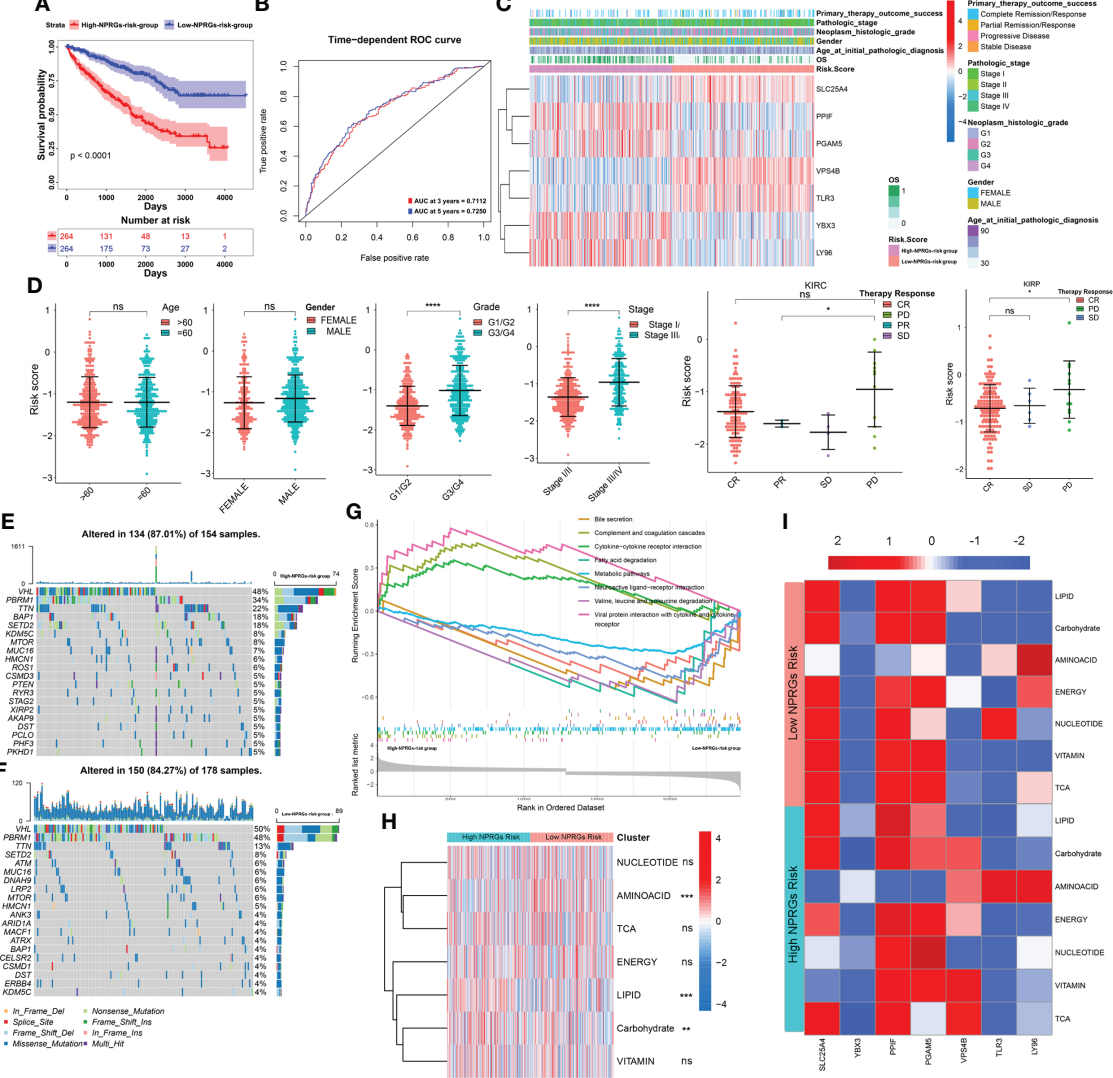
Analysis of clinical relevance, somatic mutation, mechanisms, and metabolism reprogramming based on the NPRGss-risk signature. **(A–C)** The Kaplan–Meier survival curves, ROC curve, and heatmap based on the NPRGs risk signature. **(D)** The comparison of the gender, age, stage, grade, and therapy response in the high- or low-NPRGs-risk group. **(E, F)** The waterfall diagram shows the somatic mutation situation of the top 20 genes in the high- or low-NPRGs-risk group of the TCGA-KIRC cohort. **(G)** GSEA-enrichment plot of top 10 KEGG pathways. **(H)** Heatmap shows the activity of seven metabolic signatures in the high- or low-NPRGs-risk group of the TCGA-KIRC cohort. **(I)** Heatmap displays the correlation between NPRGss and seven metabolic signatures in the high- or low-NPRGs-risk group of the TCGA-KIRC cohort, respectively. *p-value < 0.05, **p-value < 0.01, ***p-value < 0.001, ****p-value < 0.0001.

We further analyzed the clinical characteristics, potential molecular mechanism, immunity, and metabolism of TCGA-KIRC patients based on the seven NPRGss. In the KIRC cohort, the survival difference of the high- or low-NPRGs-risk group and predictive performance of NPRGs risk signature and the expression alteration of seven NPRGss were consistent with previous studies (**Figures 4A–C**). With the increase in risk score, we observed that pathological grade and tumor stage also increased (**Figure 4D**). In addition, we found that patients with progressive disease (PD) showed a high risk score, revealing that seven NPRGs signatures could predict therapy response. We further explored the distinction in genetic alteration between high- or low-NPRGs-risk groups. The missense mutation, SNP, and C>T are the main type of variant classification (**Supplementary Figure S5B**). The median number of variants per sample in the high-NPRGs-risk group is 52, whereas that in the low-NPRGs group is 44. Moreover, 87.01% of samples of the high-NPRGs-risk group have a mutation, which was higher than the low-NPRGs-risk group (84.27%) (**Figures 4E, F**). In the top 20 mutated rate genes, the PBRM1’s mutation rate in the high-NPRGs-risk group (34%) was lower than that in the low-NPRGs-risk group (48%). The SETD2’ mutation rate in the high NPRGs-risk group (18%) was higher than in the low NPRGs-risk group (8%). BAP1 had a high mutation rate (18%) in the high-NPRGs-risk group, whereas the low-NPRGs-risk group had not. Previous literature has shown that these gene mutations were associated with KIRC survival and response to immunotherapy (25). These results revealed that the mutation burden in the high-NPRGs-risk group is higher than that in the low-NPRGs-risk group. We further explored the underlying molecular mechanisms of NPRGs risk signature. The DEGs in the high-NPRGs-risk group compared to the low-NPRGs-risk group were calculated and subjected to clusterProfiler R-package (**Supplementary Table S6**). We found that multiple immune-related pathways or GO terms were highly enriched in the high-NPRGs-risk group, such as complement and coagulation cascades, humoral immune response, and antigen binding (**Figure 4G**; **Supplementary Figure S5C**, detail in **Supplementary Table S7**). However, metabolism-related pathways or GO terms are highly enriched in low-NPRGs risk. Furthermore, the activity of lipid, carbohydrate, and amino acid pathways was significantly different in high- or low-NPRGs-risk groups (**Figures 4H, I**).

### Construction of a nomogram model based on the clinical signature and NPRGss

**Figure 5:**
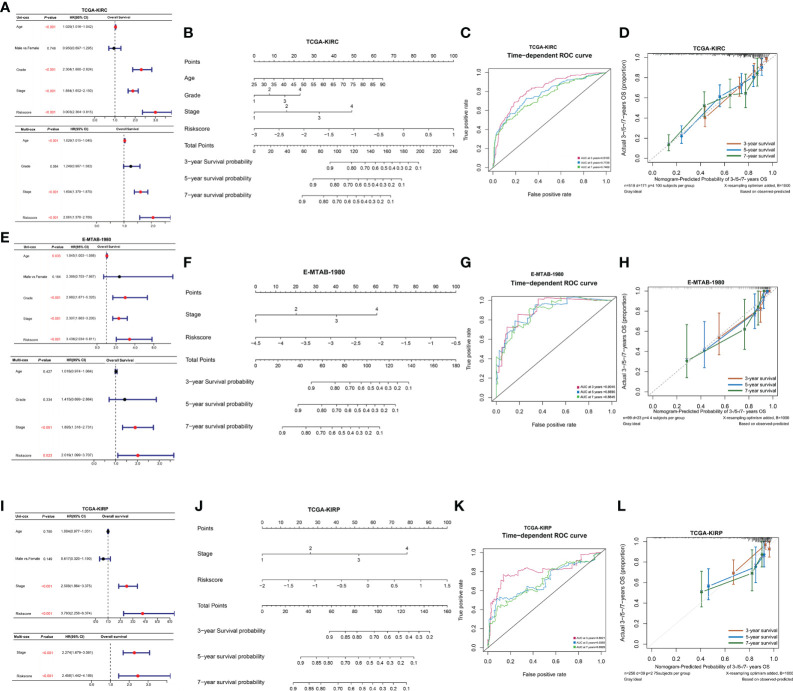
Clinical model construction of NPRGss-risk signature. **(A)** Univariate/multivariate Cox regression analysis regarding the OS in the TCGA-KIRC cohorts. **(B)** Nomogram model in TCGA-KIRC cohort. **(C)** The ROC curves of the nomogram model in the TCGA-KIRC cohort. **(D)** Calibration curves of OS probability over 3, 5, and 7 years based on nomogram model in TCGA-KIRC cohort. **(E–H)** Univariate/multivariate Cox regression analysis, nomogram construction, ROC curve analysis, and calibration curve analysis in the E-MTAB-1980 cohort. **(I–L)** Univariate/multivariate Cox regression analysis, nomogram construction, ROC curve analysis, and calibration curve analysis in the TCGA-KIRP cohort, ns, no significance.

To further explore the independent prediction value of NPRGs risk signature, the univariate or multivariate Cox regression analysis was conducted utilizing age, gender, pathological stage, histological grade, and NPRGs risk signature in the TCGA-KIRC cohort. The results pointed out that NPRGs risk signature (HR = 2.061, p-value< 0.001, multivariate Cox regression) was the independent prognostic factor for TCGA-KIRC patients (**Figure 5A**). We further used these independent prognostic factors estimated by multivariate Cox regression analysis based on the stepwise regression method to construct a nomogram model to predict the survival probability of 3/5/7 years of patients (**Figure 5B**). ROC and calibration curve analysis indicated that the model possesses a discriminative accuracy for 3-/5-/7-year survival (**Figures 5C, D**). Furthermore, DCA curves showed that the model consisting of risk score, pathological stage, age, and histological grade has the best clinical application value for patient prognosis prediction (**Supplementary Figure S5D**). We conducted the same study in the E-MTAB-1980 cohort (**Figures 5E–H**, **Supplementary Figure S5D**) and got similar results. Interestingly, the total points calculated based on the nomogram were highly consistent in predicting 3-/5-/7-year survival probability in the TCGA-KIRC and E-MTAB-1980 cohort, which further highlighted the predictive power of the prognostic model for KIRC patient prognosis. To improve the applicability of NPRGs risk signature, we also conducted the same study in the TCGA-KIRP cohort. We were excited that the results were still highly similar, which means that our signature may be applicable to other RCC tumors (**Figures 5I–L**; **Supplementary Figure S5D**).

### Prediction of targeted drugs and immune therapy response of NPRGs risk signature

Previous studies have confirmed that patients with PD showed high NPRGs risk scores. Thus, we speculate that NPRGs risk signature could predict drug therapy response. We first provided the DEGs in high- or low-NPRGs-risk groups to a drug prediction website—DeSigN (19). A connectivity score closer to 1 indicates that the drug has the most excellent efficacy in the high-NPRGs-risk group. The results suggested that patients in the high-NPRGs-risk group might benefit better when treated with etoposide, DMOG, BMS-754807, and lenalidomide (**Supplementary Figure S6A**). Further analysis showed that the estimated IC50 values of rapamycin, sorafenib, and pazopanib were lower in the low-NPRGs-risk group, revealing that the patients in low-NPRGs-risk groups may be more sensitive to these three targeted drugs (**Supplementary Figure S6B**, detail in **Supplementary Table S8**).

**Figure 6:**
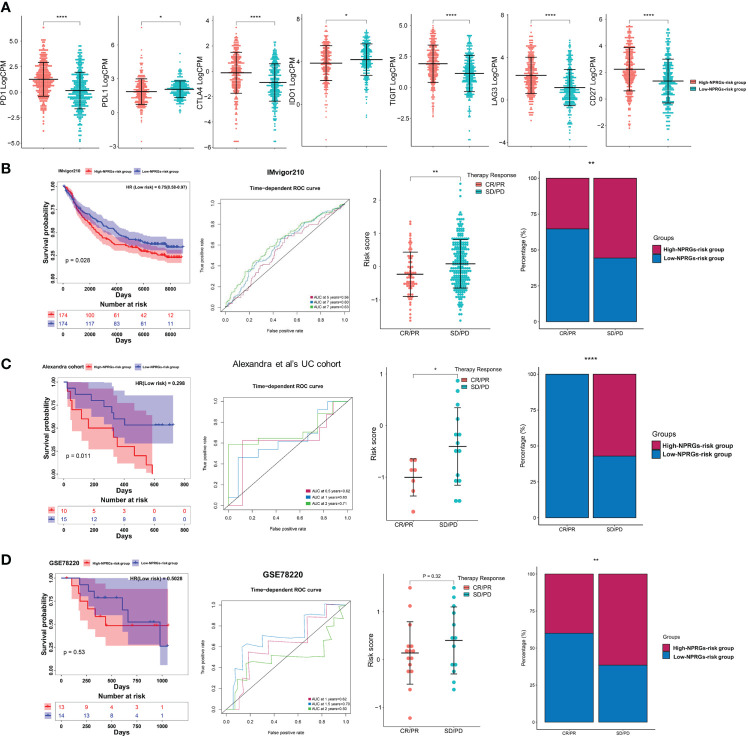
**(A)** The immune checkpoints’ expression in the high- or low-NPRGs-risk group of TCGA-KIRC dataset. **(B)** Validation of the NPRGs risk signature in the IMvigor210 cohort with the survival analysis, ROC curves analysis, and immune therapy response analysis. **(C)** Validation of the NPRGs risk signature in the UC cohort of Alexandra et al. with the survival analysis, ROC curves analysis, and immune therapy response analysis. **(D)** Validation of the NPRGs risk signature in the GSE78220 cohort with the survival analysis, ROC curves analysis, and immune therapy response analysis. CR, complete response; PR, partial response; SD, stable disease; PD, progressive disease. *p-value < 0.05, **p-value < 0.01, ****p-value < 0.0001.

We further explored the immune therapy response based on the NPRGs risk signature. We found that the activity and percentage of immune cells showed a significant difference in high- or low-NPRGs-risk groups (**Supplementary Figures 6C–E**). The immune-inhibited cell, macrophage M2, and Tregs, which have been found to promote tumor growth, showed high activity and percentage in high-NPRGs-risk groups. Furthermore, we found that the expressions of PD1, CD274, TIGIT, LAG3, and CD27 were higher in the high-NPRGs-risk group. The expressions of PDL1 and IDO1 were higher in the low-NPRGs-risk group (**Figure 6A**). These results may imply that patients in different risk groups may discrepantly respond to immunotherapy. As we did not have access to data from KIRC treated with immune therapy, we collected three external publicly available immunotherapy data (IMvigor210, GES78220, and the UC cohort of Alexandra et al.), which represent different cancer types treated with anti-PD1 therapy to verify our NPRGs risk signature and provide indirect evidence for predicting response to immunotherapy for renal cell carcinoma patients. The Kaplan–Meier curve indicated that patients in the high-NPRGs-risk group showed a poor prognosis (**Figures 6B–D**), and the NPRGs risk score showed a feasible prediction performance. In addition, we found that patients with progressive disease (PD) and stable disease (SD) showed a high NPRGs risk. Most patients in the low-NPRGs-risk groups could receive a complete response (CR) and partial response (PR). These results suggested that the patients with low NPRGs risk may not be sensitive to immunotherapy.

## Discussion

Necroptosis was found as a novel regulated cell death that shows a morphological resemblance to necrosis and a mechanistic resemblance to apoptosis (26). Necroptosis is pivotal in regulating cancer biology, including oncogenesis, immunity, metabolism, and metastasis (3). The plasma membrane rupture caused by necroptosis leads to the release of cell components, which could cause a robust inflammatory response and trigger an anticancer immune response through mediating the interaction between immune cells and cancer cells (27). Meanwhile, necroptosis was also involved in maintaining T-cell homeostasis by clearing up abnormal and excessive T cells without CASP8 (28). Metabolic reprogramming is critical for the function of carcinogenesis (29). Necroptosis could activate immune response by regulating metabolism programming (30). Hence, targeting immune metabolism based on necroptosis was considered to be a tumor therapy strategy.

Renal cell carcinoma (RCC) is considered an immunogenic tumor (31). The interaction between tumor cells and the tumor immune microenvironment (TIME) relates to the molecular drivers underlying RCC occurrence, metastasis, and recurrence. Furthermore, mutations in kidney cancer genes are implicated in many diverse aspects of cellular metabolisms, such as oxygen, glutamine metabolism, and the tricarboxylic acid (TCA) cycle, so kidney cancer has also been labeled as a metabolic disease (32). Over the past two decades, the treatment of RCC has undergone extensive alterations. Except for surgical excision, combinations of treatment of immune-checkpoint inhibitors (ICIs), mTOR pathways, and vascular endothelial growth factor (VEGF) pathway inhibition have shown remarkable efficacy in patients with metastatic RCC and gradually become the first-line therapies for patients with this disease (33, 34). However, despite the relatively high response rates to immune and target therapy for RCC, most RCC patients do not receive durable clinical benefits due to drug resistance (35). Therefore, there is an urgent need for a detailed classification of RCC patients to screen suitable patients for immune therapy. Previous research revealed that necroptosis might be the main pathway of cell death based on the TNF-targeted therapy in RCC (8). Inhibiting necroptosis could promote RCC cell proliferation (7). Activating necroptosis combining with immunotherapy has been found to increase anti-tumor immune responses (36, 37). At present, more and more genes associated with necroptosis have been discovered, and drugs targeting necroptosis-related targets are also being developed in renal cancer (38). However, the influence or concrete mechanism of necroptosis on prognosis and immune infiltration in RCC still needs to be explored further.

Previous research has indicated the importance of necroptosis-related genes in KIRC and constructed the risk models to predict the prognosis of patients (9). However, these models did not validate through multiple KIRC cohorts and guide clinical therapy. Our study determined 12 NPRGss in KIRC by analyzing the public database. Then, we further screened seven NPRGss to construct a novel NPRGs risk signature by LASSO Cox regression analysis. The model could predict the prognosis and therapy response of the patients with KIRC and has a good prediction effect for KIRP. Previous studies have indicated that specific subtypes of tumors may respond differently to immunotherapy (39). In multiple immune therapy cohorts, our NPRGs risk signature showed a good prediction performance. The SD/PD patients showed a higher NPRGs risk score. CR/PR patients showed lower NPRGs risk signature. These anti-PD1 data come from different types of cancer cohorts, which also provide potential evidence that the NPRGs risk signature could screen the specific type of RCC patients who are sensitive to immune therapy. For low-NPRGs-risk patients, aggressive use of anti-PD1 therapy may be effective for long-term survival. For high-NPRGs-risk patients, the aggressive use of current therapies is accompanied by the early consideration of other better options, such as increased susceptibility to immunotherapy by targeting NPRGss. In high-NPRGs-risk groups, YBX3, LY96, PGAM5, and PPIF showed a higher expression than low-NPRGs-risk groups. These results indicated that high co-expression of these four genes could cause poor prognosis in KIRC and KIRP patients, which is also suitable for KIRP patients. A previous study indicates that the upregulation of YBX3 increases cancer cell invasion and tumor chemoresistance (40). Overexpression of PPIF confers poor prognosis in endometrial cancer (41). Depleting PGAM5 expression inhibited tumor growth, and restoring PGAM5 expression could enhance tumor resistance (42). LY96, which is known as MD-2, targeting it therapies have been shown to prevent colon cancer growth and lung metastasis. (43). However, the concrete mechanism of these genes on tumor prognosis and immune therapy response still remains unclear. Therefore, exploring the specific mechanism of function of NPRGss in KIRC may provide a foundation for further targeted therapeutic strategies based on necroptosis and further understanding of their promoting or suppressing roles in immunotherapy response. In addition, we also observed that prognosis, stage, grade, immune checkpoint expression, immune cell activity, immune cell fraction, and metabolism signature activity were discrepant in these two cohorts. Among these, the Treg activity is higher in high-NPRGs-risk groups. Tregs are mainly responsible for regulating the immune system, maintaining self-antigens’ tolerance, and preventing autoimmune disease (44). Elevated infiltration levels of Treg cells were correlated with a worse prognosis in tumor tissues (45). There is sufficient evidence that the depletion and inhibition of Treg function could enhance anti-tumor effects (46). Tregs play an important role in immune suppression, which could control B cells and NK cells, T cells, dendritic cells (DCs), and macrophage cell–cell contact mechanisms (47). Furthermore, the M2 macrophage fraction is higher in high-NPRGs-risk groups, which have been reported to be related to worse outcomes in KIRC (48). Macrophages play a pivotal role in stimulating proliferation, angiogenesis, and metastasis in tumors (49). The sum of pre-clinical human and animal studies reveals that targeting tumor-associated macrophages (TAMs) could significantly improve the clinical efficacy of conventional and immune therapeutics (50). Metabolic reprogramming has been an area of intense research over the last decade (51). The metabolic signatures analysis showed that LIPID signature activity was significantly highly expressed in the high-NPRGs-risk group. Reprogramming of lipid metabolism is a newly recognized malignancy hallmark (52); defects in lipid metabolism induce abnormal gene expression and rewire many oncogenesis-/metastasis-related pathways (53). Therefore, interfering with lipid metabolism within the tumor may be a novel target for tumor immunotherapy (54). These results suggest that necroptosis may affect the prognosis of patients by regulating immunity and metabolism. Studying immune and metabolism dysregulation based on necroptosis may further understand the specific mechanisms of tumor progression.

Although the NPRGs risk signature is more robust for predicting prognosis and immune therapy response, there are several limitations, including the various cohorts with different cancer types or sample numbers, data format, and gene detecting technology. The retrospective collection of clinical data and lack of clinical information of the therapy cohort could cause different prediction discrepancies and instability of the model. Validating our models as an independent predictor of prognosis and therapy response required prospective studies. In addition, the molecular mechanism of NPRGss in KIRC is still unclear, and individual bioinformatic analyses still need further experimental validation.

In conclusion, our study highlighted the importance of NPRGss in KIRC and established a novel NPRGs risk signature that was verified to be an independent prognostic factor for OS in KIRC. The signature could be extended to the prognosis prediction of KIRP patients. The NPRGs risk signature can predict immunotherapeutic response, which provides a foundation for developing therapeutic strategies based on NPRGss in KIRC.

## Data availability statement

The datasets presented in this study can be found in online repositories. The names of the repository/repositories and accession number(s) can be found in the article/**Supplementary Material**.

## Author contributions

Conception and design: JL, XL, ED, ZZ, YL. Development of methodology: JL, XL, YQ. Acquisition of data: JL, XL, YQ, YL. Analysis and interpretation of data (e.g., statistical analysis, computational analysis): JL, XL, YQ. Writing, review, and/or revision of the manuscript: JL, XL, YQ, ED, ZZ. Administrative, technical, or material support: JL, XL, ED, ZZ, YL. Study supervision: ED, ZZ. All authors contributed to the article and approved the submitted version.

## Funding

This work was supported by The National Natural Science Foundation of China (NO. 22076138) and Tianjin Science and Technology planning project (NO. 21JCYBJC01750).

## Conflict of interest

The authors declare that the research was conducted in the absence of any commercial or financial relationships that could be construed as a potential conflict of interest.

## Publisher’s note

All claims expressed in this article are solely those of the authors and do not necessarily represent those of their affiliated organizations, or those of the publisher, the editors and the reviewers. Any product that may be evaluated in this article, or claim that may be made by its manufacturer, is not guaranteed or endorsed by the publisher.

## Supplementary material

Supplementary Figure 1 Expression alteration of NPRGss in KIRC. **(A)** The boxplots show the expression of 12 NPRGss in the TCGA-KIRC tumor and adjacent normal tissues. The significance level between the two groups was calculated with a t-test. **(B)** Immunohistochemistry of the NPRGss in KIRC and adjacent normal tissues.

Supplementary Figure 2 The Kaplan-Meier survival curves showed the relationship between the individual NPRGs’s expression in TCGA-KIRC. The log-rank test was used to calculate the P-value.

Supplementary Figure 3 Cross-talk and mechanisms of 7 NPRGss in KIRC. **(A)** The correlation diagrams show the correlation among the expression of 7 NPRGss in TCGA-KIRC. Red represents the positive correlations, and blue represents the negative correlations. The size of the point showed a significant level. **(B)** The PPI network plot showed the cross-talk of NPRGss in protein levels. **(C)** The PPI network diagram shows the cell components, molecular functions, biological processes, and KEGG that are highly correlated with the expression of 7 NPRGss. **(D)** The heatmap shows the correlations between the activity score of cancer-related hallmark pathways and the NPRGss’ expression.

Supplementary Figure 4
**(A)** The interaction between NPRGss, miRNAs, and LncRNAs. **(B)** The interaction between NPRGss and the regulators of RNA modification.

Supplementary Figure 5
**(A)** Validation of the NPRGss-risk signature in TCGA-KIRC cohort divided by clinicopathologic features with the Kaplan-Meier analysis. **(B)** The variant situations in the high- or low-NPRGss-risk group of the TCGA-KIRC cohort. **(C)** Bar plot showing the top10 significant enrichment terms of cell component, biological progression, and molecular functions in the high- or low-NPRGss-risk group of TCGA-KIRC cohort. **(D)** DCA curves of 3, 5, and 7 years based on the clinical models in the TCGA-KIRC cohort, E-MTAB-1980 cohort, and TCGA-KIRP cohort.

Supplementary Figure 6
**(A)** Drug prediction outcome is utilizing the DeSigN website. **(B)** The estimated IC50 of chemotherapy and targeted therapy drugs in the high- or low-NPRGss-risk group. **(C)** The percentage of immunity cells in the high- or low-NPRGss-risk group. **(D)** The activity scores of adaptive immunity cells in the high/low-NPRGs-risk group. **(E)** The activity scores of innate immunity cells in the high/low-NPRGs-risk group.
