# Supplementary figures and images for: A risk signature based on necroptotic-process-related genes predicts prognosis and immune therapy response in kidney cell carcinoma
Source: Front Immunol. 2022 Sep 16;13:922929. doi: 10.3389/fimmu.2022.922929 (PMC9524857; doi:10.3389/fimmu.2022.922929)

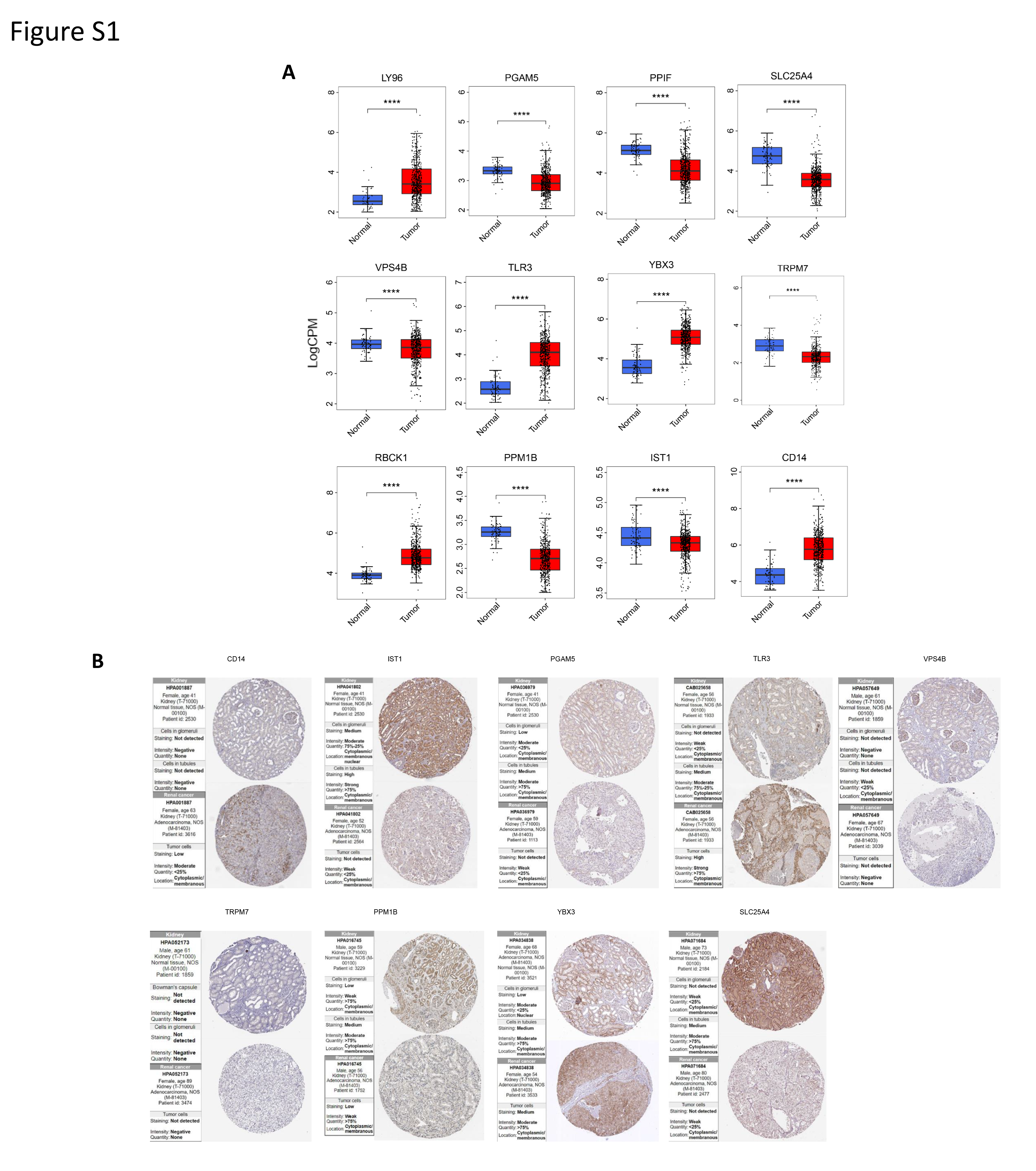

Supplement: Supplementary Figure 1 — Expression alteration of NPRGss in KIRC. (A) The boxplots show the expression of 12 NPRGss in the TCGA-KIRC tumor and adjacent normal tissues. The significance level between the two groups was calculated with a t-test. (B) Immunohistochemistry of the NPRGss in KIRC and adjacent normal tissues. [file Image_1.tif]

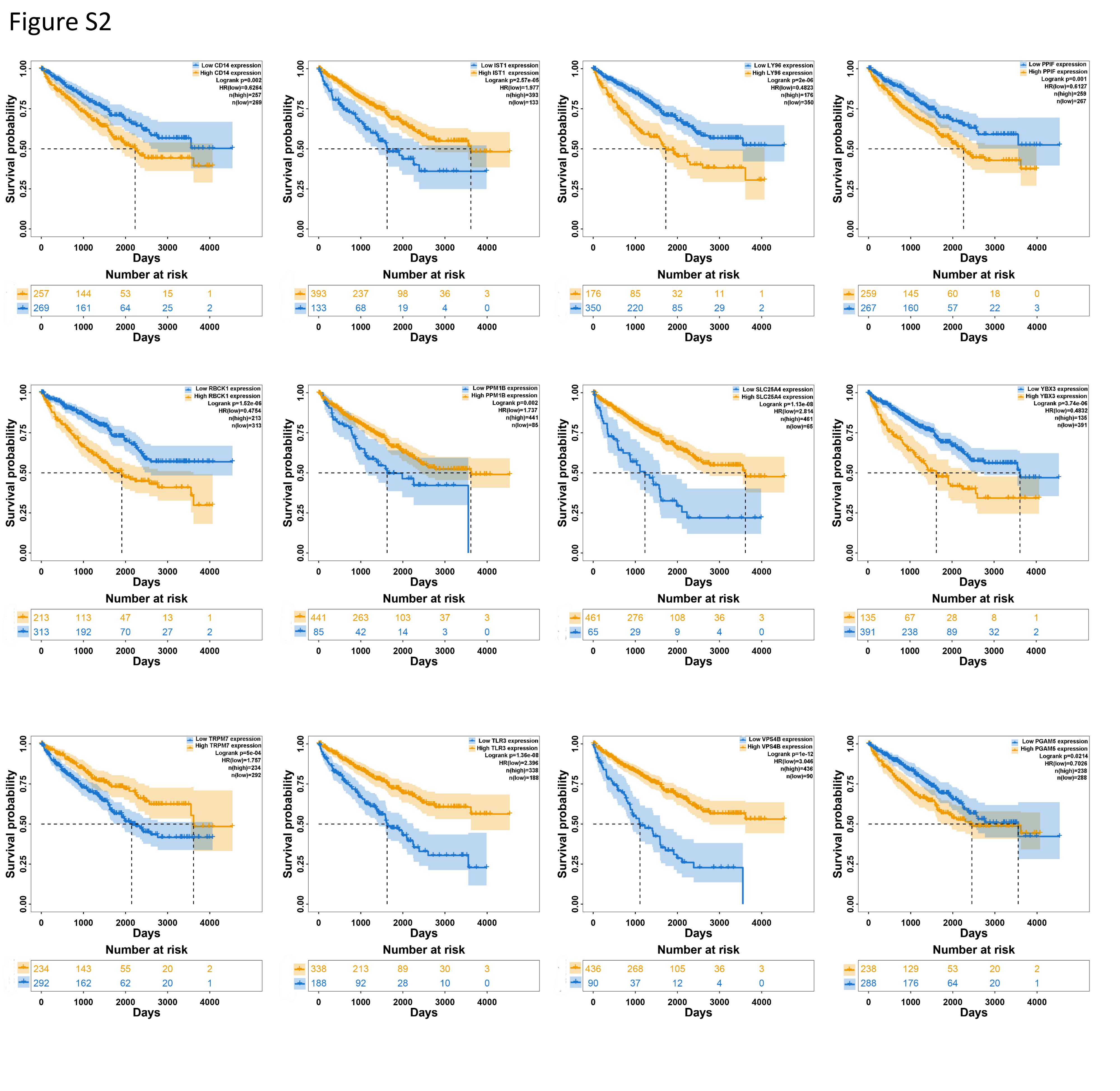

Supplement: Supplementary Figure 2 — The Kaplan-Meier survival curves showed the relationship between the individual NPRGs’s expression in TCGA-KIRC. The log-rank test was used to calculate the P-value. [file Image_2.tif]

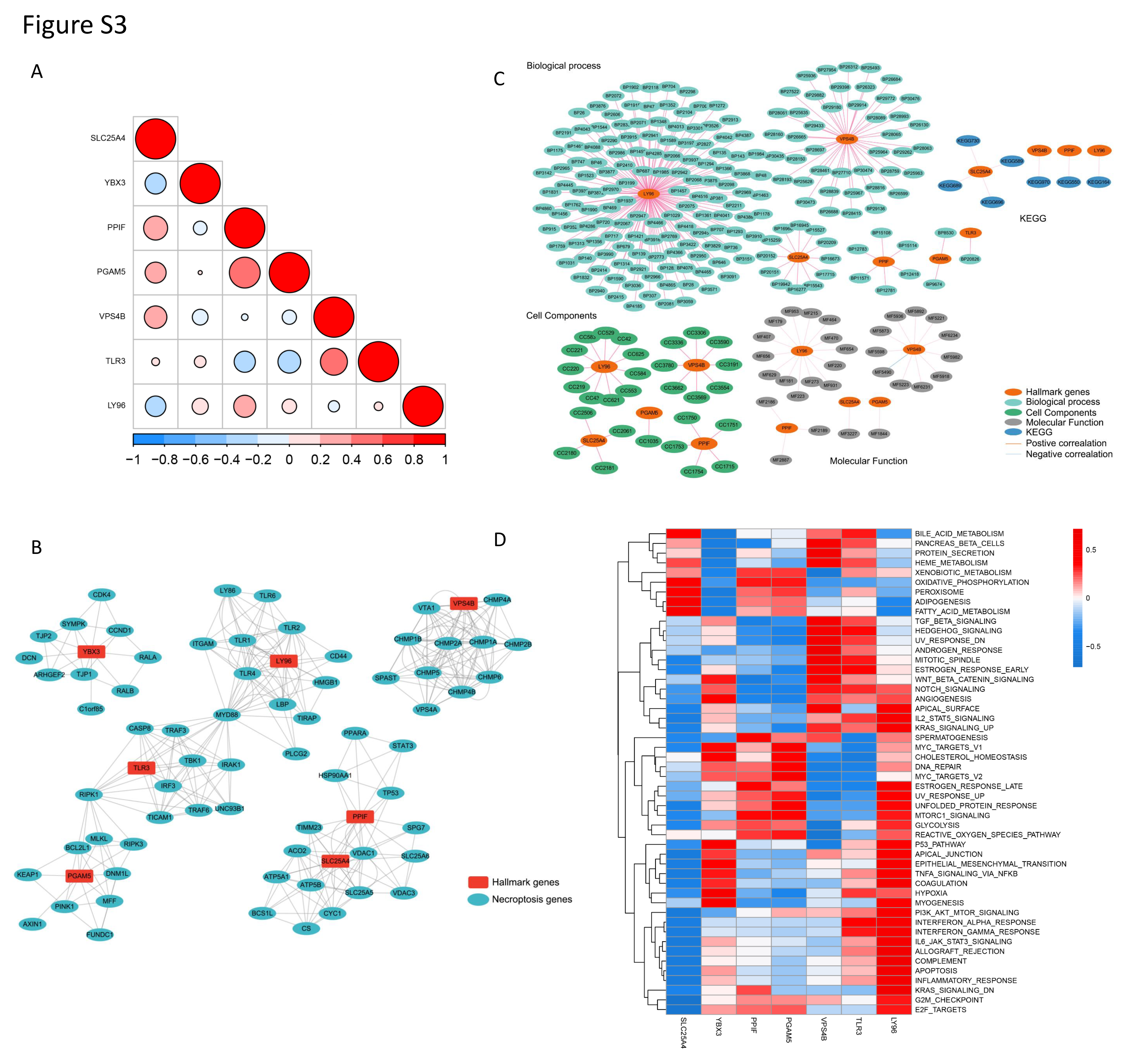

Supplement: Supplementary Figure 3 — Cross-talk and mechanisms of 7 NPRGss in KIRC. (A) The correlation diagrams show the correlation among the expression of 7 NPRGss in TCGA-KIRC. Red represents the positive correlations, and blue represents the negative correlations. The size of the point showed a significant level. (B) The PPI network plot showed the cross-talk of NPRGss in protein levels. (C) The PPI network diagram shows the cell components, molecular functions, biological processes, and KEGG that are highly correlated with the expression of 7 NPRGss. (D) The heatmap shows the correlations between the activity score of cancer-related hallmark pathways and the NPRGss’ expression. [file Image_3.tif]

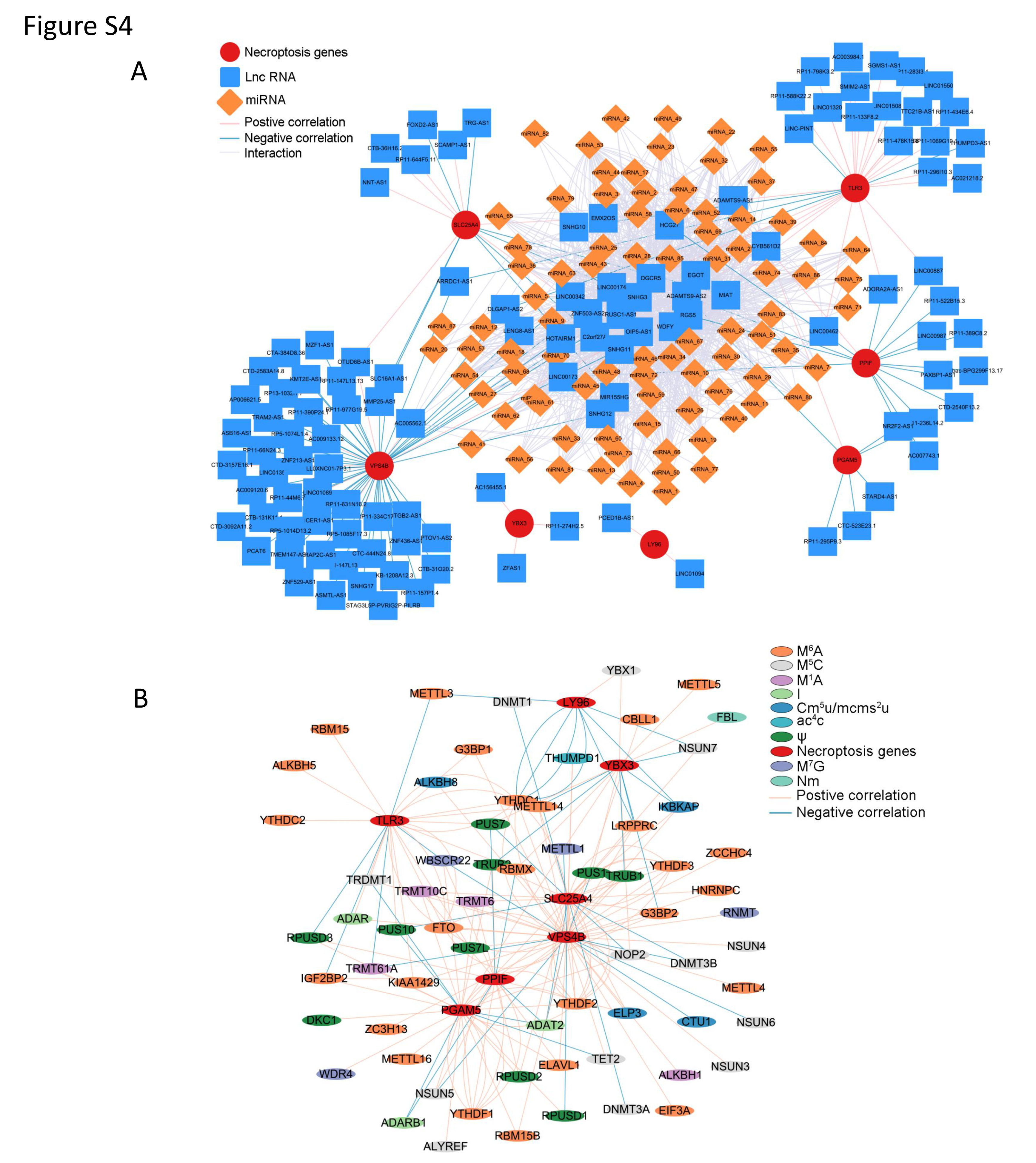

Supplement: Supplementary Figure 4 — (A) The interaction between NPRGss, miRNAs, and LncRNAs. (B) The interaction between NPRGss and the regulators of RNA modification. [file Image_4.tif]

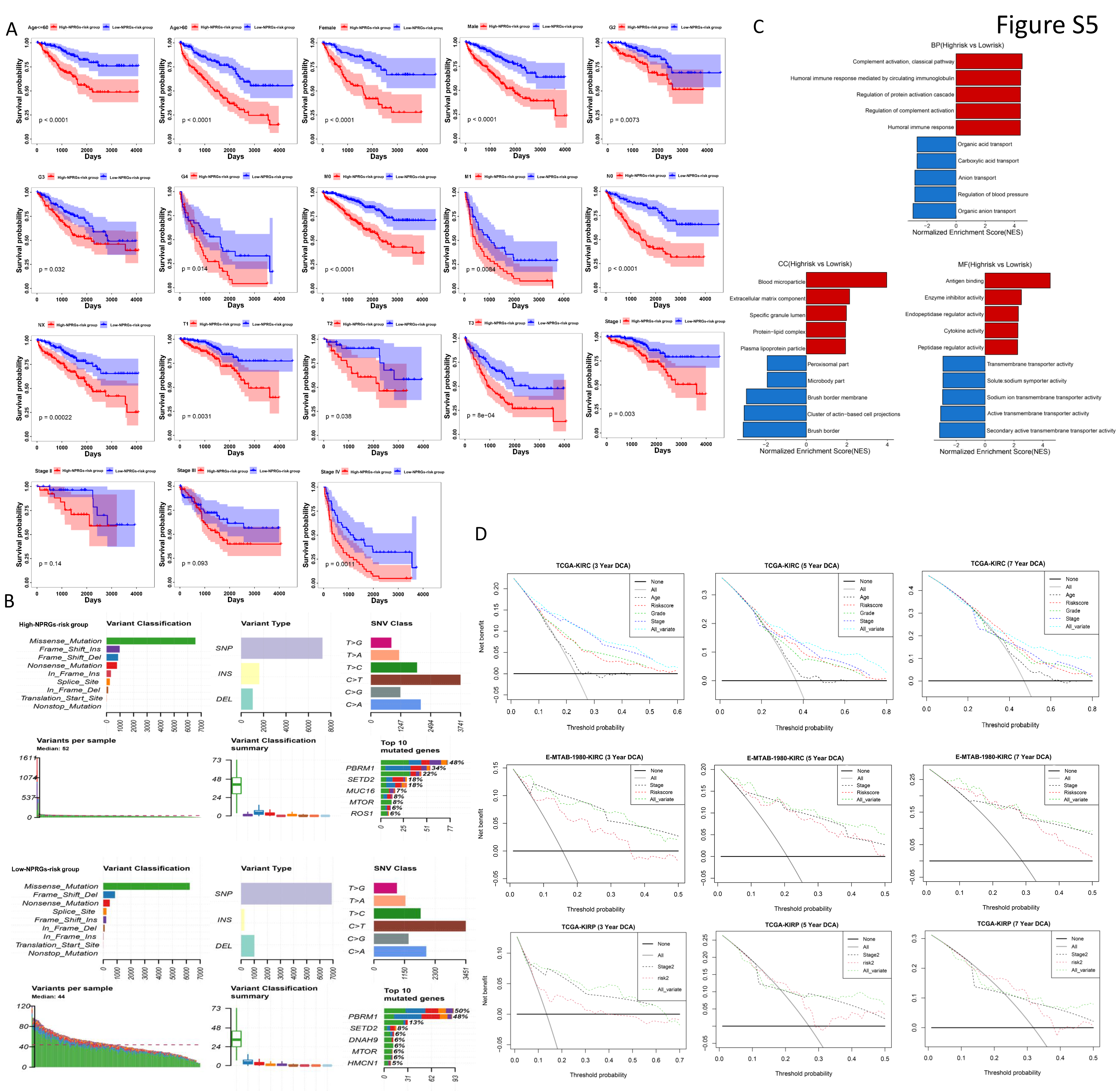

Supplement: Supplementary Figure 5 — (A) Validation of the NPRGss-risk signature in TCGA-KIRC cohort divided by clinicopathologic features with the Kaplan-Meier analysis. (B) The variant situations in the high- or low-NPRGss-risk group of the TCGA-KIRC cohort. (C) Bar plot showing the top10 significant enrichment terms of cell component, biological progression, and molecular functions in the high- or low-NPRGss-risk group of TCGA-KIRC cohort. (D) DCA curves of 3, 5, and 7 years based on the clinical models in the TCGA-KIRC cohort, E-MTAB-1980 cohort, and TCGA-KIRP cohort. [file Image_5.tif]

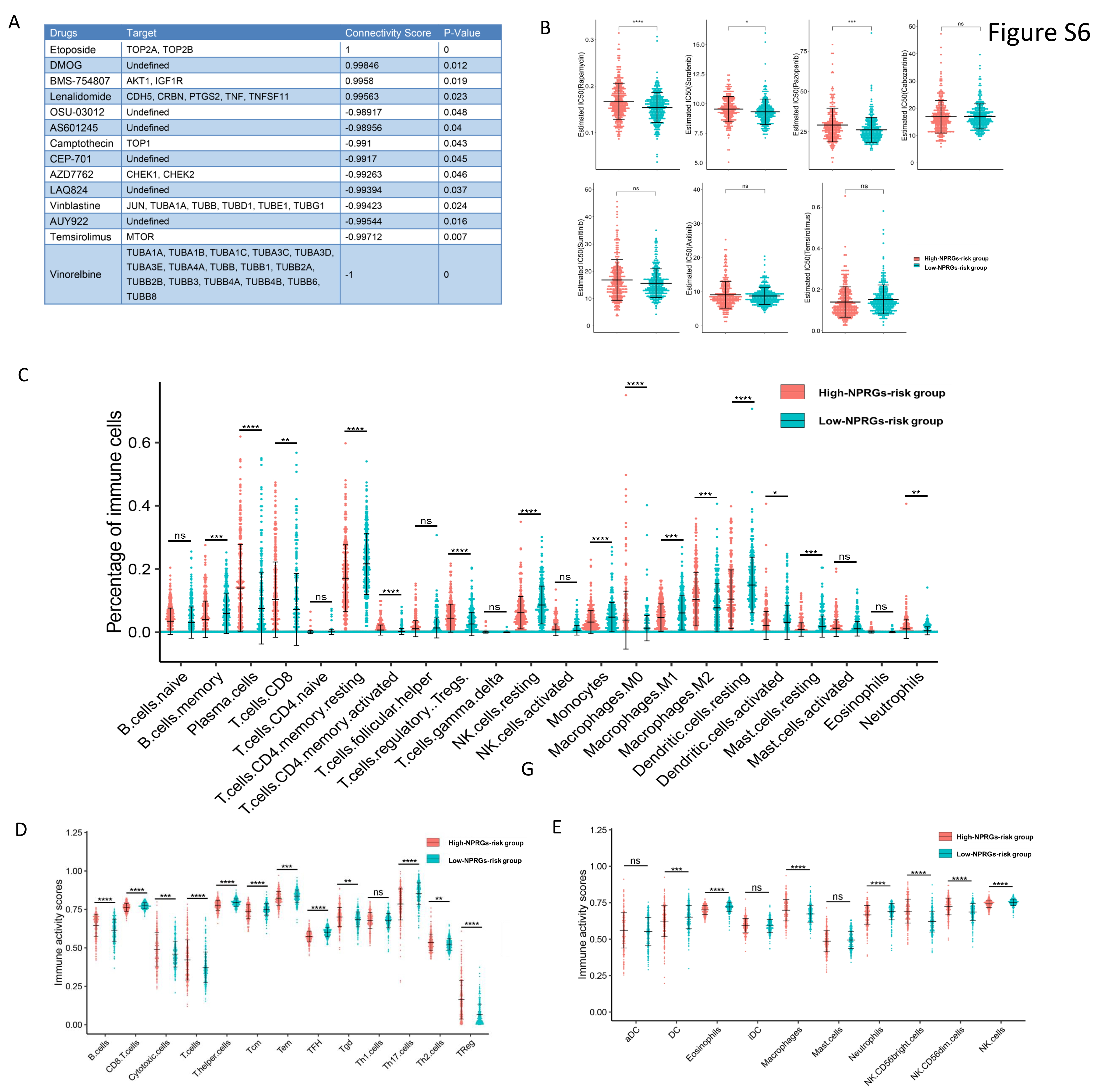

Supplement: Supplementary Figure 6 — (A) Drug prediction outcome is utilizing the DeSigN website. (B) The estimated IC50 of chemotherapy and targeted therapy drugs in the high- or low-NPRGss-risk group. (C) The percentage of immunity cells in the high- or low-NPRGss-risk group. (D) The activity scores of adaptive immunity cells in the high/low-NPRGs-risk group. (E) The activity scores of innate immunity cells in the high/low-NPRGs-risk group. [file Image_6.tif]
